# American Medical Association

**Published:** 1861-05

**Authors:** 


AMERICAN MEDICAL ASSOCIATION.—Just as we were going to press we received a letter from Dr. N. S. Davis, Chairman of the Committee of Arrangements, stating that, in consequence of the distracted condition of the country, the meeting of the American Medical Association would be postponed until June, 1862.

